# Causal relationship between circulating immune cells and diabetic nephropathy: A Mendelian randomization study

**DOI:** 10.1097/MD.0000000000043182

**Published:** 2025-08-22

**Authors:** Tao Wang, Chun Huang, Yuxia Mo, Jinshuai Li, Xiangjian Wu, Xiaoyan Fu, Yimin Hu, Geping Wu, Chunfeng Yang, Minfang Li, Sheng Chen

**Affiliations:** a Shenzhen Traditional Chinese Medicine Hospital, Shenzhen, Guangdong, China; b The Fourth Clinical Medical College, Guangzhou University of Chinese Medicine, Shenzhen, Guangdong, China; c School of Life Sciences, Beijing University of Chinese Medicine, Beijing, China.

**Keywords:** circulating leukocytes, circulating lymphocyte subtypes, DN, GWAS, Mendelian randomization analysis

## Abstract

This study explores the causal relationship between circulating immune cells and diabetic nephropathy (DN) progression using Mendelian randomization. We selected instrumental variables from genome-wide association study summary statistics, which included datasets of circulating leukocytes, flow cytometric subgroups of lymphocytes, and DN. Applying the standard Mendelian randomization approach and refining it with sensitivity analysis and horizontal pleiotropy examination, we found a significant causal association between increased eosinophil counts and a higher risk of DN (odds ratio: 1.21, 95% confidence interval: 1.07–1.37, *P* = .00027), which remained robust after further analysis. However, no causal link was observed between lymphocyte flow cytometric subgroups and DN. These findings suggest that elevated eosinophil counts may contribute to the progression of DN, potentially advancing to end-stage renal disease. The lack of a causal relationship between lymphocyte subtypes and DN underscores the need for larger scale genome-wide association study data for further validation. This research provides important causal evidence for considering eosinophil counts as a potential biomarker in DN diagnosis and management.

## 1. Introduction

Diabetic nephropathy (DN) is a prominent complication of diabetes, characterized by persistent microalbuminuria, a gradual decline in renal function, and kidney interstitial fibrosis.^[1]^ In type 1 diabetes patients, the incidence of DN ranges from 25% to 40%, and in those with type 2 diabetes, it is between 5% and 40%.^[2]^ Epidemiological studies show that nearly half of DN patients may progress to end-stage renal disease without effective intervention.^[3]^ Traditionally, DN pathogenesis is attributed to direct kidney damage due to prolonged hyperglycemia, hemodynamic alterations, abnormal growth factor and cytokine activation, and genetic predispositions.^[4]^ However, recent research has linked DN to inflammatory and immune responses, particularly involving macrophages, lymphocytes, and mast cells.^[5]^ These cells exacerbate renal damage and fibrosis by releasing inflammatory mediators.^[6]^ Macrophages, as the primary infiltrating leukocytes, are linked to the decline in renal function and the development of glomerulosclerosis.^[7]^ In the early stages of DN, the accumulation of T cells, B cells, neutrophils, and dendritic cells significantly influences disease progression.^[8]^ Among them, neutrophils exacerbate inflammation and kidney damage by releasing neutrophil extracellular traps (NETs). NETs, composed of DNA and neutrophil enzymes, can damage glomerular endothelial cells, promoting cell necrosis and dysfunction.^[9]^ Neutrophil activation also increases the production of reactive oxygen species and triggers the NLRP3 inflammasome, further intensifying inflammation and kidney injury. Therefore, inhibiting the formation of NETs or maintaining neutrophil enzyme stability may offer new therapeutic strategies for diabetic kidney disease.^[6]^ However, current research on DN predominantly utilizes observational methods, which constrains our understanding of the causal relationship between circulating immune cells and the disease’s pathogenesis. Further research is imperative to elucidate this causative connection, thereby enriching our etiological knowledge of the condition.

The rapid and extensive advancements in genome-wide association studies (GWAS) facilitated the use of Mendelian randomization (MR) to deduce causal relationships among complex traits.^[10]^ It leverages the random assortment of genes to mitigate confounding factors, revealing the impact of these exposures on disease risk. MR, thus, uses gene variations as a tool to infer causal links between exposure and disease.^[11]^ MR, by utilizing multiple single nucleotide polymorphisms (SNPs) less affected by confounders, produces more reliable outcomes than traditional studies, effectively reducing concerns about reverse causation and confounding.^[12]^ MR utilizes genetic variation as a proxy for long-term or lifelong exposure, avoiding the errors and biases often found in observational studies. Consequently, MR is particularly suited for large-scale research to clarify the causal connections between circulating immune cells and DN. This methodology opens new avenues for enhancing DN patient care and broadens the strategic options for its prevention and treatment.

## 2. Methods

### 2.1. Data sources

The circulating leukocyte data used in our study were obtained from the blood cell consortium, which includes information from a cohort of 562,243 individuals of European descent.^[13]^ For further details, please refer to Table S1, Supplemental Digital Content, https://links.lww.com/MD/P628. This GWAS provided genetic variants associated with levels of various circulating leukocytes, including lymphocytes, monocytes, neutrophils, eosinophils, and basophils. Additionally, we analyzed the flow cytometry spectrum for lymphocyte subgroups, comprising HLA DR + natural killer (NK) cells (GWAS ID:ebi-a-GCST90001648), natural killer T absolute count cells (GWAS ID:ebi-a-GCST90001621), CD4 + regulatory T cells (Treg) (GWAS ID:ebi-a-GCST90001513), CD4 + CD8dim T cells (GWAS ID:ebi-a-GCST90001609), CD8 + T cells (GWAS ID:ebi-a-GCST90001592), and B cells (GWAS ID:ebi-a-GCST90001642), using recent GWAS summary statistics from 3757 Sardinians.^[14]^ The GWAS summary data on DN were derived from the Finnish Biobank, encompassing a cohort of 5763 individuals, including 2035 females and 3728 males. The median age at the first event was 61.26 years, with a distinction between genders: 63.76 years for males and 56.69 years for females. The diagnosis was substantiated through hospital discharge and cause of death records, relying on professional diagnoses indicated by the ICD-10 code N08.3 (https://www.finngen.fi/en).^[15]^

### 2.2. Selection of instrumental variable (IV)

MR studies rely on several fundamental premises: (a) the selected IV needs to have a robust correlation with the exposure, and this relationship should be free from the influence of any confounders. (b) the IV should not be linked to confounding factors that may affect both exposure and outcome; (c) the IV should influence the outcome solely through the exposure.^[6]^ Adhering to these principles, we selected SNPs with *P*-values <5 × 10^−8^ as IVs for circulating leukocytes. In our study, we referred to the data from 1000 Genomes Project for establishing a cutoff for linkage disequilibrium, setting it at an *r*^2^ value <0.001 across a physical distance of 1000 kb. For lymphocyte subgroup GWAS, which are of a relatively medium scale, we adopted a more lenient *P*-value of 5 × 10^−6^ and a clustering threshold (*R*^2^ < 0.1 within 500 kb).^[16]^ The effectiveness of the IVs was evaluated using the F-statistic $\left(F=\frac{N-K-1}{K}\times \frac{R^{2}}{1-R^{2}}\right)$. IVs with an F-statistic value below 10 were excluded to ensure that phenotypic differences between subgroups were primarily attributable to genetic variations, thereby influencing the outcomes.^[17,18]^

### 2.3. MR analysis and sensitivity tests

In our study, the primary method utilized was the inverse variance weighted (IVW) method, which facilitated the aggregation of weighted SNP causal effects to enhance the accuracy of causal inference.^[18,19]^ For assessing sensitivity, the Mendelian randomization Egger regression (MR-Egger) method was employed to estimate true causal parameters and evaluate the overarching causal relationship.^[20]^ Additionally, the weighted median (WM) method was implemented. This method can yield robust causal effect estimates, assuming at least 50% of the IVs are valid, significantly reducing the risk of type I errors.^[21]^ Moreover, the Mendelian randomization pleiotropy residual sum and outlier (MR-PRESSO) method was utilized for the detection and adjustment of horizontal pleiotropy outliers.^[22]^ To assess the presence of horizontal pleiotropy, we analyzed the MR-Egger intercept estimate; a nonzero intercept suggests the presence of horizontal pleiotropy.^[23]^ In cases where horizontal pleiotropy is detected by either method, the causal analysis using summary effect estimates test is further applied to confirm its presence.^[24]^ Heterogeneity, which violates the IV assumption, was examined using Cochran Q test to ascertain its existence.^[19,25]^ Our approach also included a leave-one-out sensitivity analysis, which involved sequentially removing 1 SNP at a time from the analysis to reevaluate the causal effect. This step was crucial for identifying outlier SNPs and bolstering the reliability of our analysis. Finally, considering the various analyses carried out in our research, we adopted an adjusted significance level of 0.05 divided by 6, based on the Bonferroni method.

Additionally, In this reverse MR analysis, we applied the same selection criteria for IVs as used for circulating leukocytes. Building upon this foundation, we proceeded with an exhaustive bidirectional MR analysis focused on outcomes related to the exposure. The objective of this analysis is to investigate the presence of potential bidirectional causal relationships, thereby reinforcing confidence in the robustness of our study findings.

## 3. Results

### 3.1. Circulating leukocytes and DN

In this MR analysis, we included 6 types of leukocytes, each represented by 171 to 454 SNPs (Table S2, Supplemental Digital Content, https://links.lww.com/MD/P628). Furthermore, the F-statistics of these SNPs were all above 10, confirming their robustness as powerful instruments.

**Figure 1.**
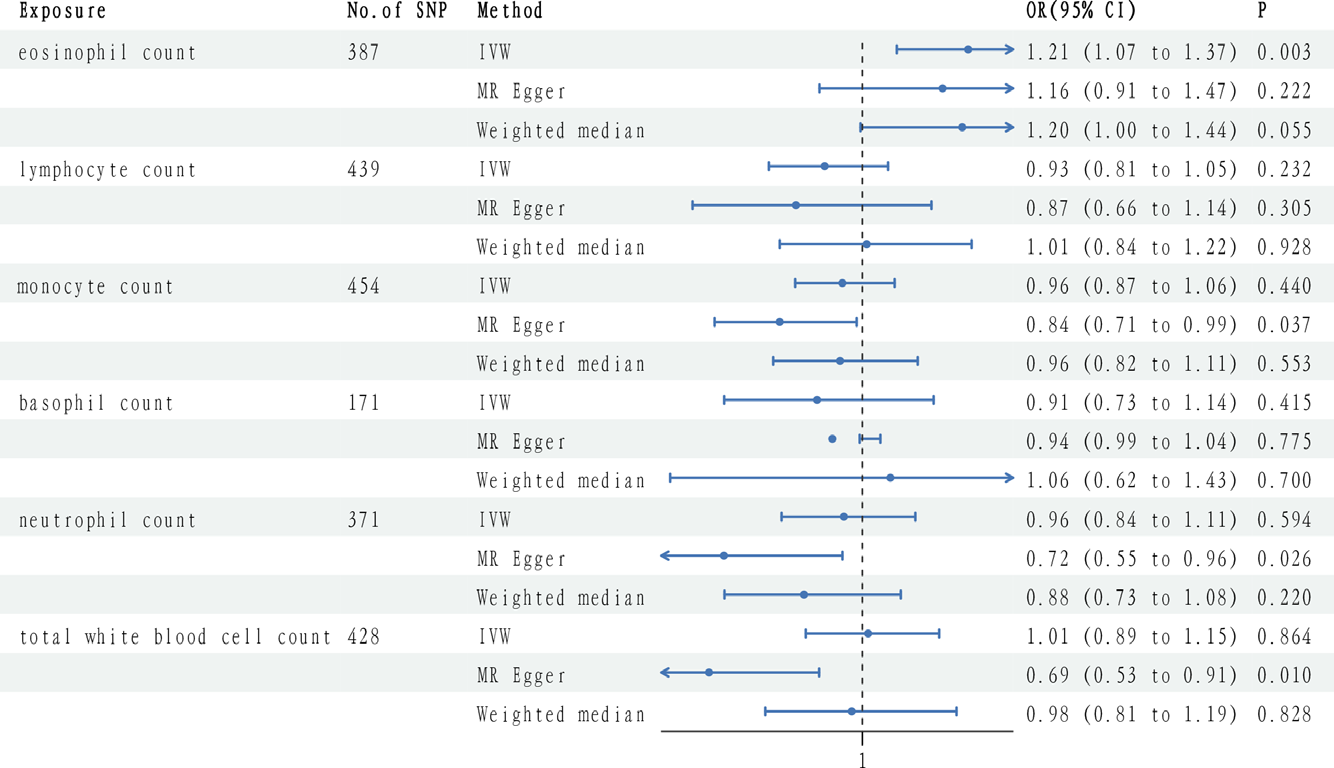
Forest plots to visualize causal effects of circulating leukocytes on the diabetic nephropathy risk. CI = confidence intervals, OR = odds ratios.

Our study results, as shown in Figure 1, reveal the causal relationship between circulating leukocyte counts and susceptibility to DN. Specifically, an increase of 1 standard deviation in eosinophil count is associated with a 21% increased risk of DN (odds ratio [OR]: 1.21, 95% confidence interval [CI]: 1.07–1.37, *P* = .00027), a result that was confirmed through the IVW method. To minimize the likelihood of type I errors and ensure the reliability of the results, we applied the Bonferroni correction. After correction, the association between eosinophil count and DN risk remained significant. This was further supported by the WM analysis (OR: 1.20, 95% CI: 0.99–1.44, *P* = .0547) and the MR-Egger method (OR: 1.16, 95% CI: 0.91–1.47, *P* = .2219), although neither of these methods yielded statistical significance. Despite the lack of significance in the WM and MR-Egger methods, the significant result from the IVW analysis provides strong evidence for a causal relationship between eosinophil count and DN. No significant associations were found between other circulating blood cells and susceptibility to DN (Table S3, Supplemental Digital Content, https://links.lww.com/MD/P628).

**Figure 2.**
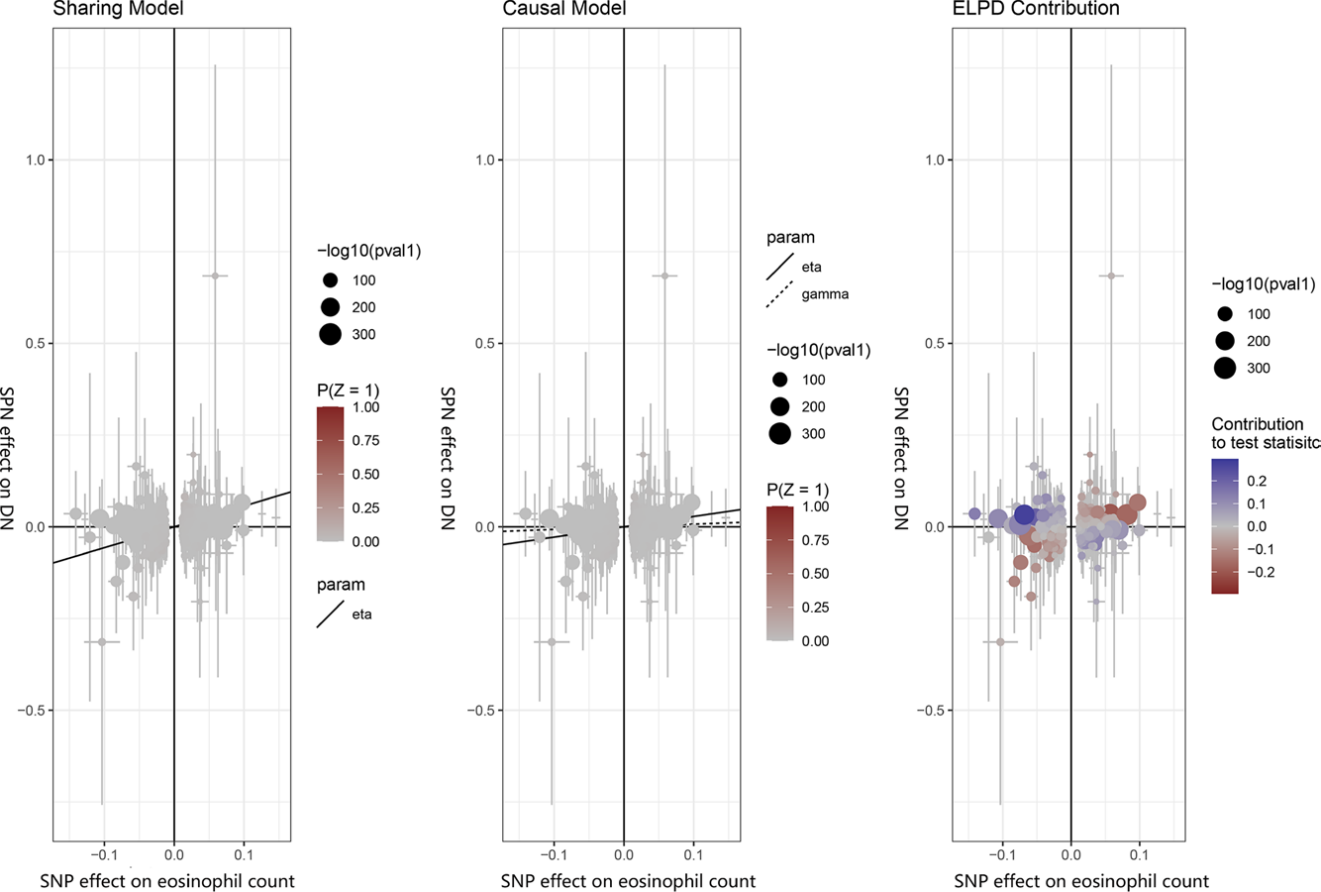
Scatter plot for the CAUSE analysis of the genetically predicted effect of circulating leukocytes on diabetic nephropathy. The ELPD Contribution diagram depicts visually the contribution of each single nucleotide polymorphism (SNP) to the CAUSE test statistic. The *P*-values of SNPs are negatively log-transformed, and larger circles represent SNPs with lower *P*-values, indicating stronger associations between genetic variants and exposure (represented on the x-axis). Those SNPs that contribute more to the causal model are depicted in red, while those that contribute more to the sharing model are depicted in blue. DN = diabetic nephropathy, SNP = single nucleotide polymorphism.

Regarding eosinophil count, while no pleiotropy was detected (Table S5, Supplemental Digital Content, https://links.lww.com/MD/P628 and Fig. S2, Supplemental Digital Content, https://links.lww.com/MD/P627), heterogeneity was observed as indicated by Cochran Q test (Table S4, Supplemental Digital Content, https://links.lww.com/MD/P628). This necessitated the use of the IVW random effects model for analysis. The MR-PRESSO test, conducted on eosinophil count with a significance threshold of *P* < .01, continued to reveal persistent horizontal pleiotropy even after the exclusion of outlier SNPs, specifically rs2713548, rs4652560, rs492430, rs6999452, and rs9266319 (Table S6, Supplemental Digital Content, https://links.lww.com/MD/P628). However, the causal analysis using summary effect estimates method, as shown in Figure 2, did not identify indications of horizontal pleiotropy (Table S7, Supplemental Digital Content, https://links.lww.com/MD/P628). Additionally, the leave-one-out plot (Fig. S1, Supplemental Digital content, https://links.lww.com/MD/P627) and the funnel plot (Fig. S3, Supplemental Digital Content, https://links.lww.com/MD/P627) indicated that there were no significant outlier SNPs, highlighting the strength and reliability of our analytical approach. The above results can be found in Tables S4 to S7, Supplemental Digital Content, https://links.lww.com/MD/P628.

### 3.2. DN and eosinophil count

In this reverse MR analysis, we included a total of 10 SNPs (Table S8, Supplemental Digital Content, https://links.lww.com/MD/P628). Based on this, we employed the MR-PRESSO method and excluded the following outlier SNPs: rs10804330, rs115048884, rs506770, rs59377618, rs689, rs9270891, and rs9273363. Despite the exclusion of these outlier SNPs, a pleiotropic effect remained evident (Table S9, Supplemental Digital Content, https://links.lww.com/MD/P628). Interestingly, no pleiotropy was detected in the pleiotropy test (see Table S10, Supplemental Digital Content, https://links.lww.com/MD/P628). The Cochran Q test results indicated significant heterogeneity among the SNPs analyzed by both the IVW and MR-Egger methods (Table S11, Supplemental Digital Content, https://links.lww.com/MD/P628), leading us to opt for the IVW random effects model. Ultimately, utilizing the results after outlier exclusion for MR analysis, we found no causal relationship between DN and eosinophil count (see Table S12, Supplemental Digital Content, https://links.lww.com/MD/P628).

### 3.3. Circulating lymphocyte subtypes and diabetic nephropathy

**Figure 3.**
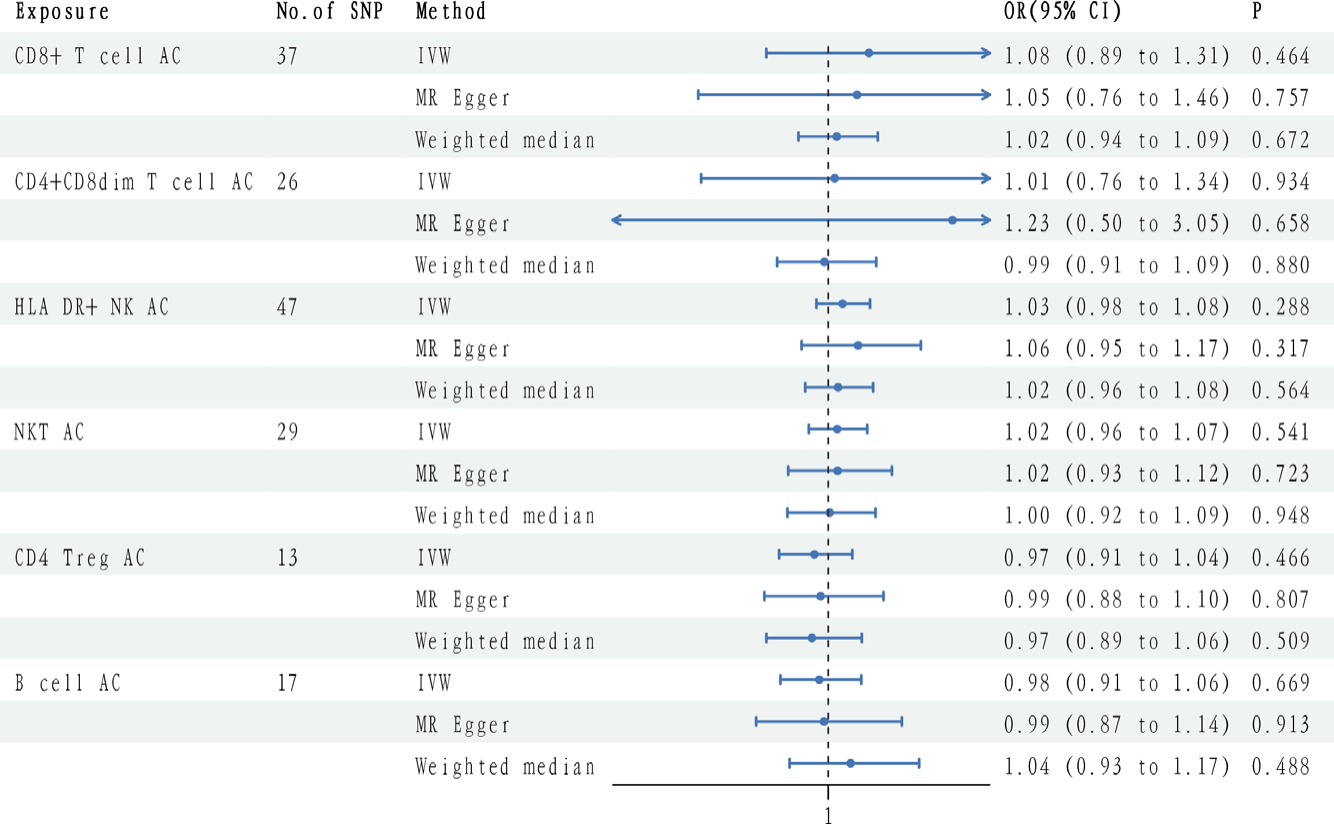
Forest plots to visualize causal effects of circulating lymphocyte subtypes on the diabetic nephropathy risk. CI = confidence intervals, OR = odds ratios.

In exploring the potential causal relationship between lymphocyte subtypes and the risk of DN, we performed additional MR analysis focusing on DN and various immune cells. The results, shown in Figure 3, suggest that lymphocyte subtypes have no causal effect on DN (Table S13, Supplemental Digital Content, https://links.lww.com/MD/P628).

## 4. Discussion

This study provides genetic evidence through MR analysis supporting a positive causal relationship between eosinophil counts and DN, while concurrently establishing that no causal relationship exists between DN and lymphocyte subtypes. Furthermore, the results from reverse MR analysis, which tested whether DN influences eosinophil counts, were not significant. This finding reinforces the causal direction established by the forward MR analysis, indicating that the relationship between eosinophil counts and DN is not driven by reverse causality. Furthermore, we conducted multiple sensitivity tests to ensure the robustness of our findings and to address potential biases due to pleiotropic effects. Our study extends previous investigations by examining the genetic basis of circulating leukocytes in DN pathogenesis, with particular emphasis on eosinophil counts.^[26]^ The integration of flow cytometric data from the Sardinian cohort with Finnish Biobank DN records provides a comprehensive assessment of immune cell involvement in DN development.^[27]^

Eosinophils, multifunctional white blood cells, play a pivotal role in various pathophysiological processes, including allergic reactions, infections, autoimmune diseases, and malignancies.^[28]^ While their pathogenic mechanisms in diseases like asthma^[29]^ and atherosclerosis^[30]^ have been extensively studied, the role of eosinophils in renal diseases remains less explored. Notably, eosinophil aggregates have been observed in the interstitium across a wide range of kidney diseases, such as DN, membranous nephropathy, IgA nephropathy, focal segmental glomerulosclerosis, and membranoproliferative glomerulonephritis.^[28–30]^ A retrospective cohort study in Japan indicated that these interstitial eosinophil aggregates are prevalent in various kidney diseases, correlating with severe chronic histological changes and a poor renal prognosis. Employing the least absolute shrinkage and selection operator method revealed that blood eosinophil count is a robust indicator for predicting interstitial eosinophil aggregation. This is particularly significant in patients with advanced chronic kidney disease, where a higher blood eosinophil count is strongly associated with increased risks of renal outcomes, underscoring the critical role of interstitial eosinophil aggregates in renal disease prognosis.^[31]^ Furthermore, these aggregates are linked to enhanced interstitial fibrosis and tubular atrophy.^[32]^

In the related literature, it has been noted that eosinophil count is negatively correlated with glomerular filtration surface density and inversely proportional to the total filtration surface per glomerulus. This implies that an increase in eosinophil levels may lead to a decline in filtration function, potentially resulting in kidney dysfunction.^[33]^ Additionally, higher HbA1c levels have been observed in patients with increased eosinophil accumulation, indicating a potential link between poor diabetes management and eosinophil accumulation in the kidneys. It is speculated that uncontrolled blood sugar levels or advanced glycation end-products might stimulate this accumulation, triggering specific regulatory responses, such as the differential secretion of preformed cytokines by eosinophils. This, in turn, could activate local inflammatory responses, elucidating the observed association between eosinophil fraction and both glomerular basement membrane width and glomerular filtration surface density. Consequently, we propose that inadequate glycemic control and advanced glycation end-products might contribute to glomerular damage (e.g., glomerular basement membrane thickening) and increased eosinophil accumulation, instigating specific regulatory responses (like cytokines and reactive oxygen species) and provoking local inflammatory reactions (such as hematuria, proteinuria, and deteriorating renal function).^[34]^ The eosinophil cationic protein (ECP), a major component of secondary eosinophil granules known for its toxicity to various parasites, bacteria, and viruses,^[35]^ has been shown to have significant correlations in DN patients. Specifically, urinary ECP to urea creatinine ratio is significantly correlated with renal function, systemic inflammation, glomerular lesions, and eosinophilic infiltration. Furthermore, ECP to urea creatinine ratio is linked to adverse renal outcomes, suggesting that urinary ECP could serve as a biomarker reflecting the extent of pathological changes in DN and assist in predicting disease progression. Nevertheless, its role as a biomarker requires further validation in larger-scale studies.^[34]^

Research has shown that in patients with type 2 diabetes mellitus-related diabetic nephropathy (T2D-DN), there is a notable downregulation of B cells, while Th1 cell subtypes, Treg, and monocytes are significantly increased.^[36]^Tregs are thought to influence inflammatory responses and may impact insulin sensitivity in DN patients, although the precise mechanisms remain a topic of debate. Compared to those in good health, T2D patients exhibit a notable decrease in peripheral blood NK cell count,^[37]^ yet the specific role of NK cells in DN has not been fully elucidated.^[38]^ Our study suggests no causal link between lymphocyte subgroups and DN, challenging the findings of previous research. We speculate that earlier studies might have been limited by small sample sizes, lack of prospective research and secondary cohort validation, and the inability to account for the effects of oral hypoglycemic drugs or insulin treatment. Additionally, the smaller sample size in the second GWAS may have led to a fewer number of standard IVs being identified, highlighting the need for further validation using larger-scale GWAS summary statistics.

In light of this, we acknowledge that while MR offers a powerful tool for reducing confounding and exploring causal relationships, it is not without its limitations. Primarily, our findings are most applicable to European populations, which restricts the universality and applicability of our discoveries globally. Therefore, it is imperative for future research to include a broader spectrum of ethnicities and geographical backgrounds to enhance the representativeness of the results. Additionally, studies involving non-European populations would help clarify potential ethnic differences in the causal relationships observed. Secondly, causal inference might be influenced by the quality and quantity of the IVs chosen, implying that our conclusions could be limited by the availability of genetic variation information. Future research should aim to expand genetic databases and improve the identification of suitable IVs, particularly those relevant to underrepresented populations. Furthermore, MR presupposes that the IVs are unaffected by confounders, a condition difficult to fully satisfy in practice, potentially leading to uncertainties in the outcomes. Future work should incorporate sensitivity analyses to evaluate the robustness of the findings and explore methods to mitigate any remaining bias from unmeasured confounders. Lastly, the study did not thoroughly validate the robustness of the causal relationships across different age and gender groups, necessitating future research to focus on these potential heterogeneities. Exploring subgroup-specific causal effects could offer more precise insights and aid in tailoring interventions for diverse demographic groups. Recognizing these limitations is not only critical for the interpretation of our study but also provides direction for future research avenues.

## 5. Conclusion

This study highlights a direct causal relationship between the increased count of eosinophils in the blood and the elevated risk of DN. This insight not only provides a new perspective on the risk factors associated with DN but also outlines a critical direction for future research. Subsequent studies should aim to uncover the biological role of eosinophils in the progression of DN and delineate the specific pathways through which they exert their influence. By delving into these mechanisms, we will be able to offer a solid scientific foundation for the development of preventative and therapeutic strategies against DN, thereby offering hope to patients.

## Acknowledgments

We are thankful for the publicly available GWAS summary data and extend our appreciation to the researchers and participants of these GWAS studies for their contributions.

## Author contributions

**Conceptualization:** Tao Wang, Chun Huang, Jinshuai Li, Sheng Chen.

**Data curation:** Tao Wang, Chun Huang, Xiaoyan Fu, Yimin Hu, Geping Wu, Chunfeng Yang, Minfang Li, Sheng Chen.

**Formal analysis:** Tao Wang, Chun Huang, Jinshuai Li, Xiangjian Wu, Xiaoyan Fu, Yimin Hu, Geping Wu, Chunfeng Yang, Minfang Li.

**Funding acquisition:** Yuxia Mo, Sheng Chen.

**Investigation:** Jinshuai Li.

**Methodology:** Tao Wang, Yimin Hu, Geping Wu, Chunfeng Yang, Minfang Li.

**Resources:** Tao Wang.

**Software:** Tao Wang, Chun Huang, Xiangjian Wu, Xiaoyan Fu.

**Supervision:** Yuxia Mo.

**Validation:** Chun Huang, Jinshuai Li.

**Visualization:** Tao Wang.

**Writing – original draft:** Tao Wang.

**Writing – review & editing:** Tao Wang, Sheng Chen.

## Supplementary Material


