# Supplementary material for: Causal relationship between circulating immune cells and diabetic nephropathy: A Mendelian randomization study
Source: Medicine (Baltimore). 2025 Aug 22;104(34):e43182. doi: 10.1097/MD.0000000000043182 (PMC12385090; doi:10.1097/MD.0000000000043182)

Figure S1: Figure S1: MR Leave-One-Out Sensitivity Analysis for Eosinophil Count on Diabetic Nephropathy (DN) SNPs:single nucleotide polymorphisms.


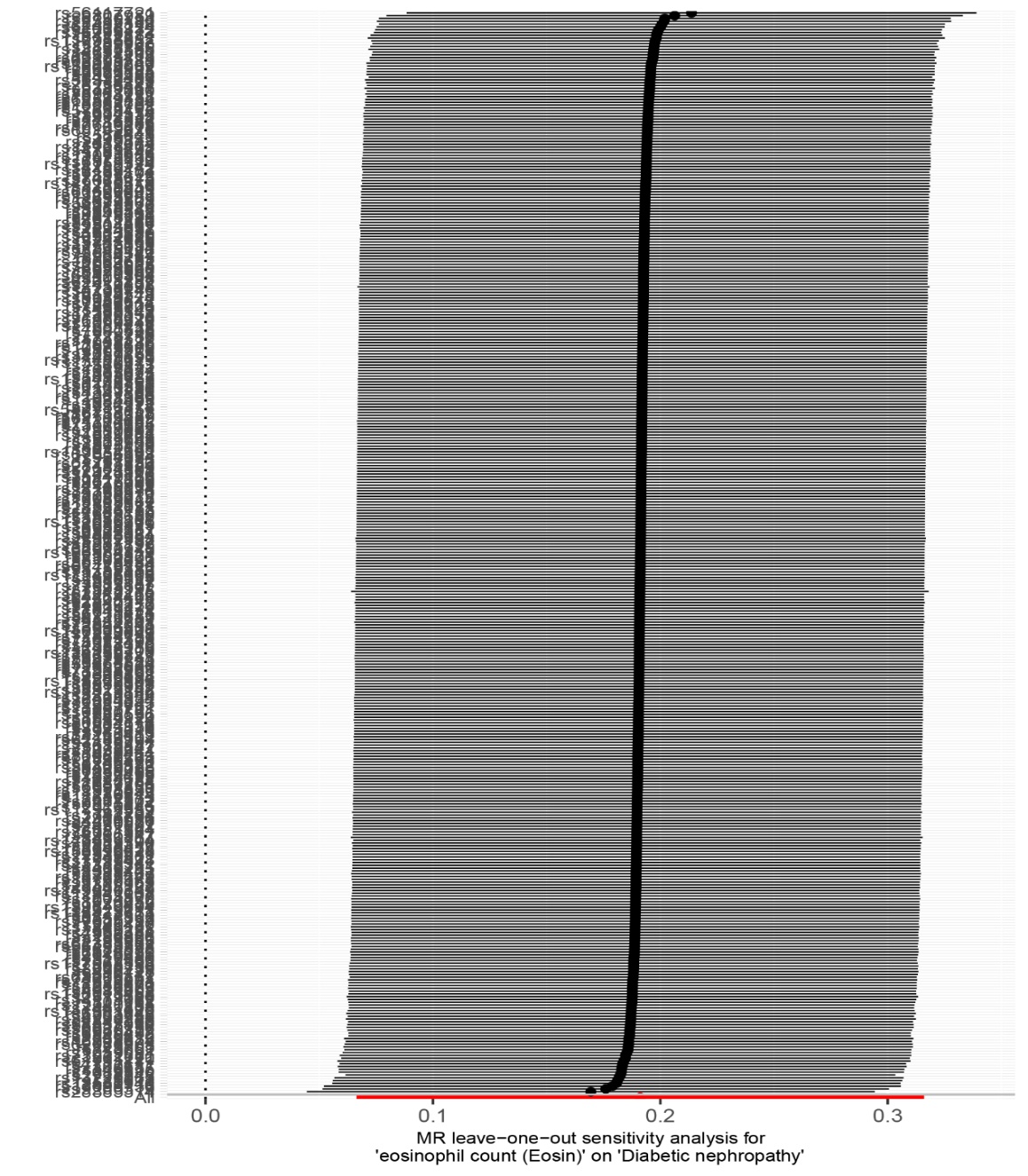


Figure S2: Scatter Plot Illustrating the Impact of Eosinophil Count on the Odds Ratio for DN. IVW: Inverse Variance Weighted.MR-Egger: Mendelian Randomization Egger Regression.WM: Weighted Median


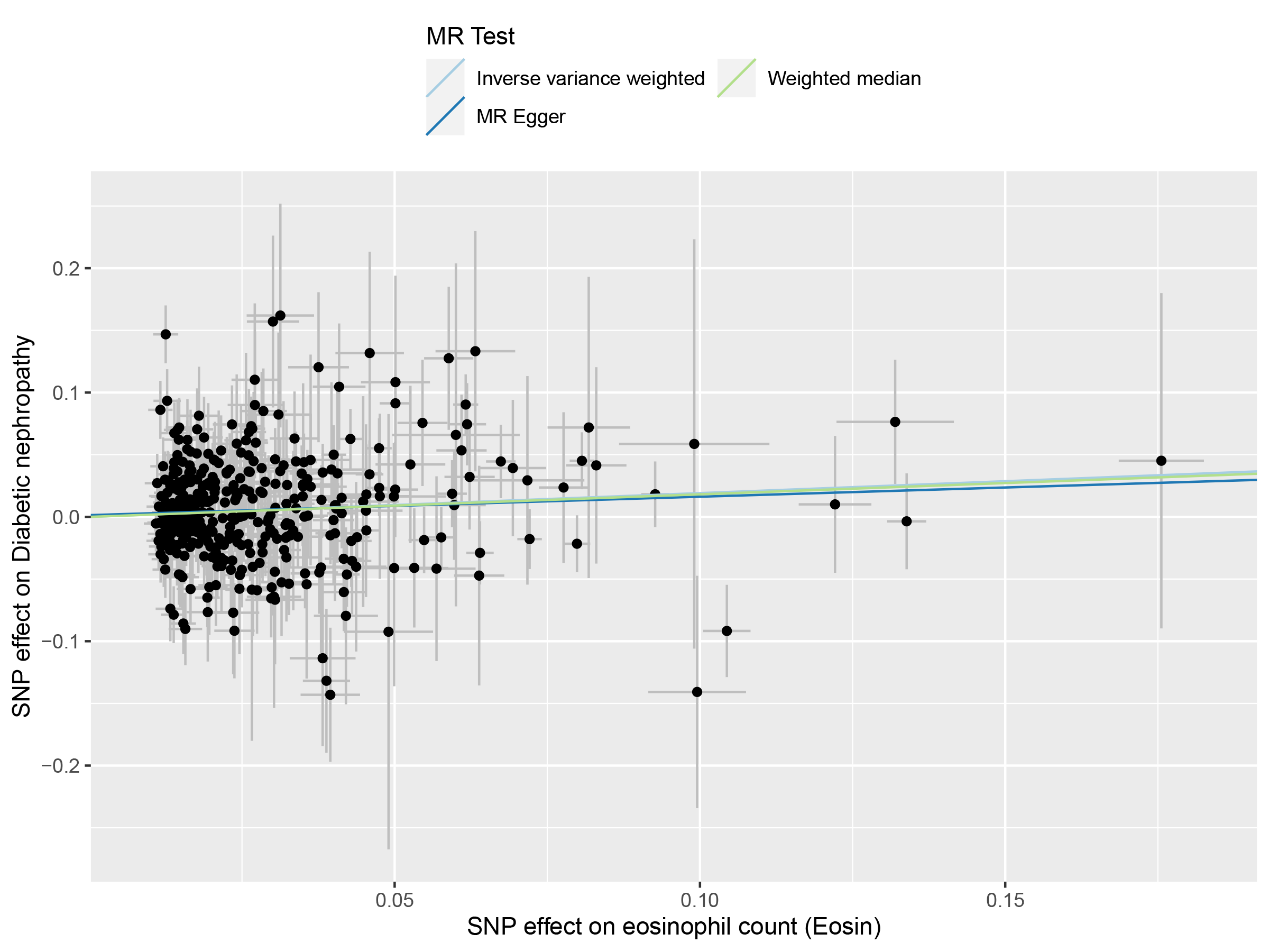


Figure S3:Funnel Plot Assessing the Effect of Eosinophil Count on DN IVW: Inverse Variance Weighted.MR-Egger: Mendelian Randomization Egger Regression.


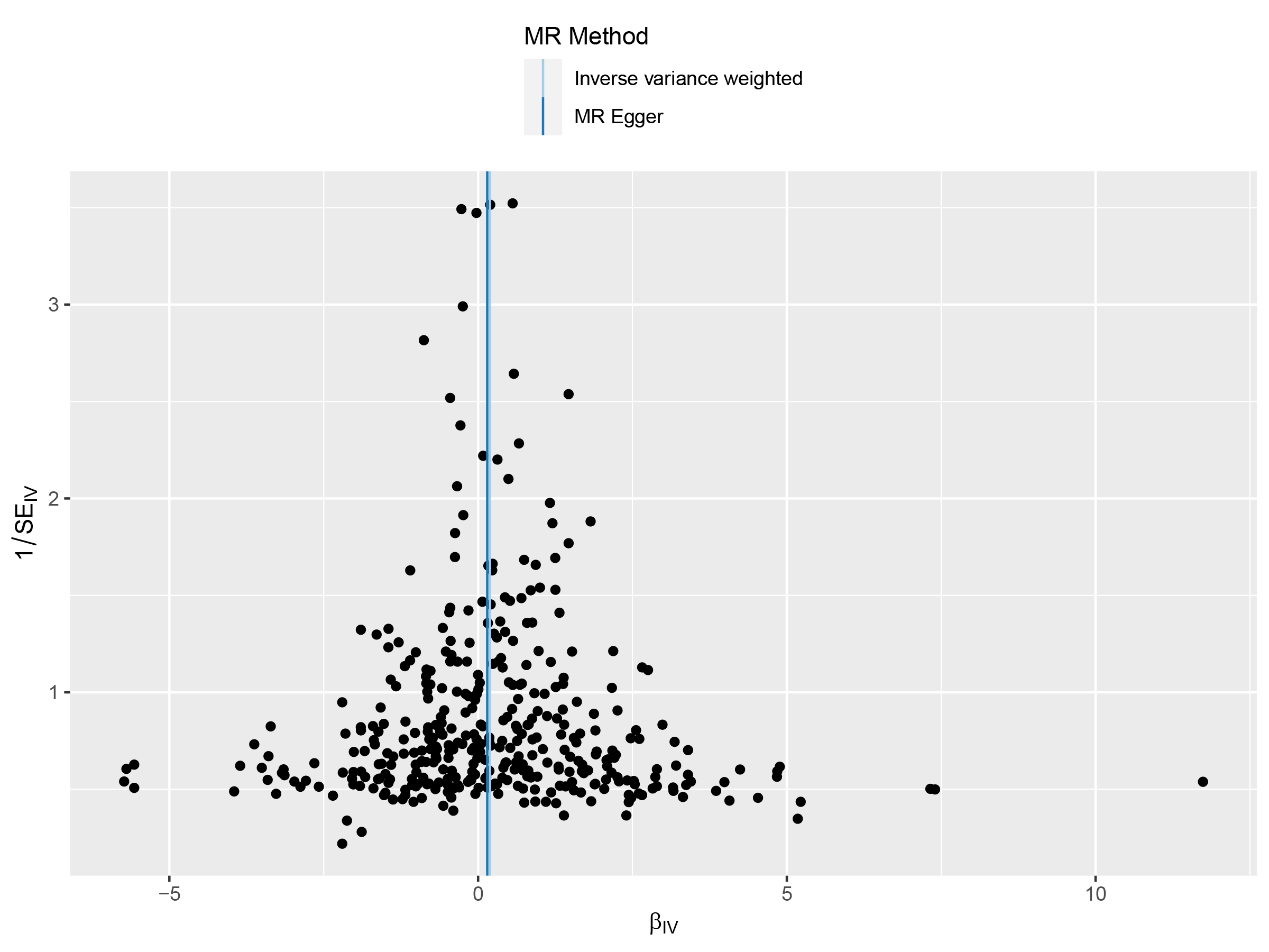

Supplement: Supplementary file 2 [file medi-104-e43182-s002.docx]
